# Supplementary figures and images for: Medium-term impacts of the waves of the COVID-19 epidemic on treatments for non-COVID-19 patients in intensive care units: A retrospective cohort study in Japan
Source: PLoS One. 2022 Sep 26;17(9):e0273952. doi: 10.1371/journal.pone.0273952 (PMC9512181; doi:10.1371/journal.pone.0273952)

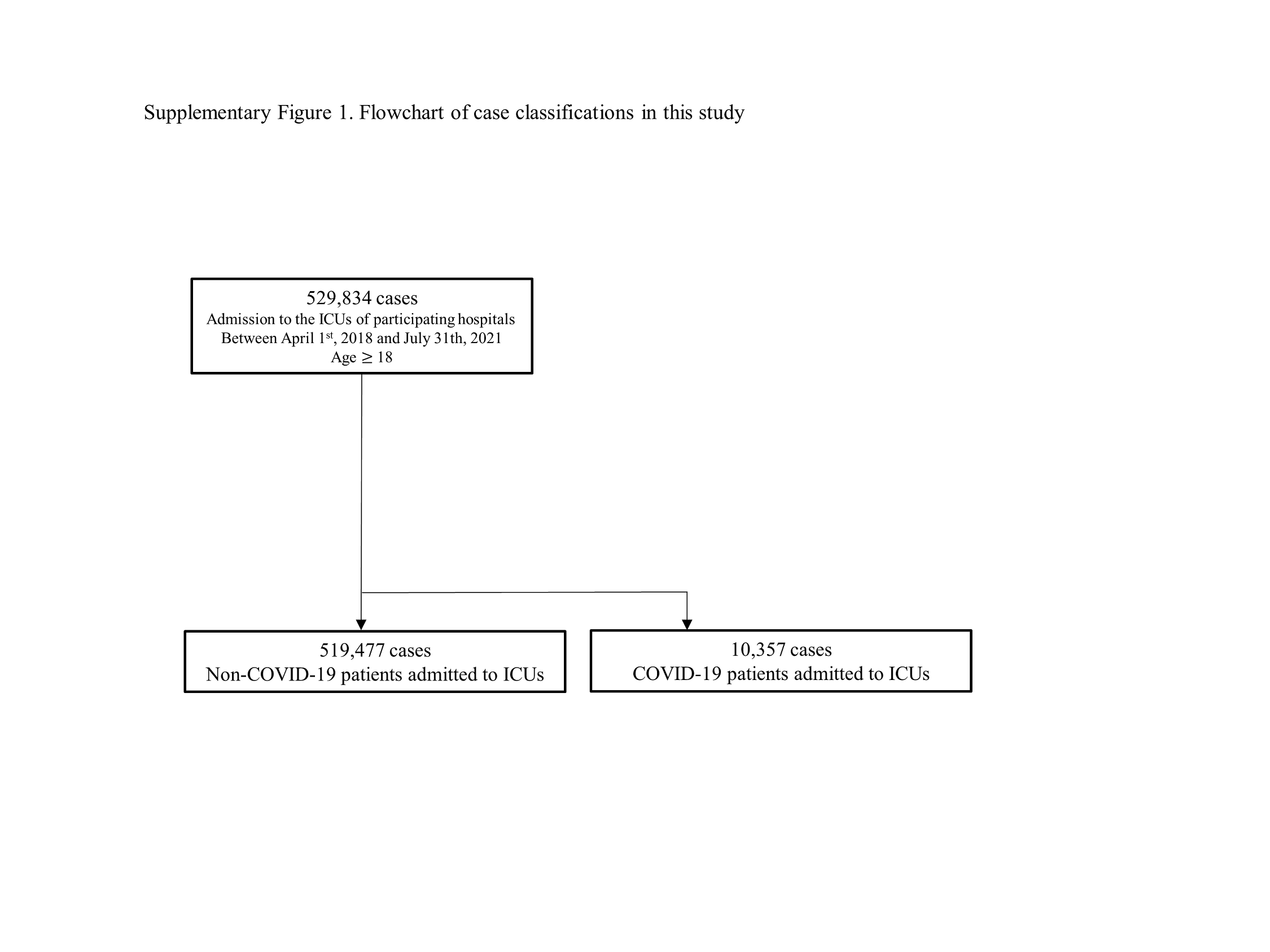

Supplement: S1 Fig — ICU, intensive care unit; COVID-19, Coronavirus disease 2019. (TIF) [file pone.0273952.s001.TIF]

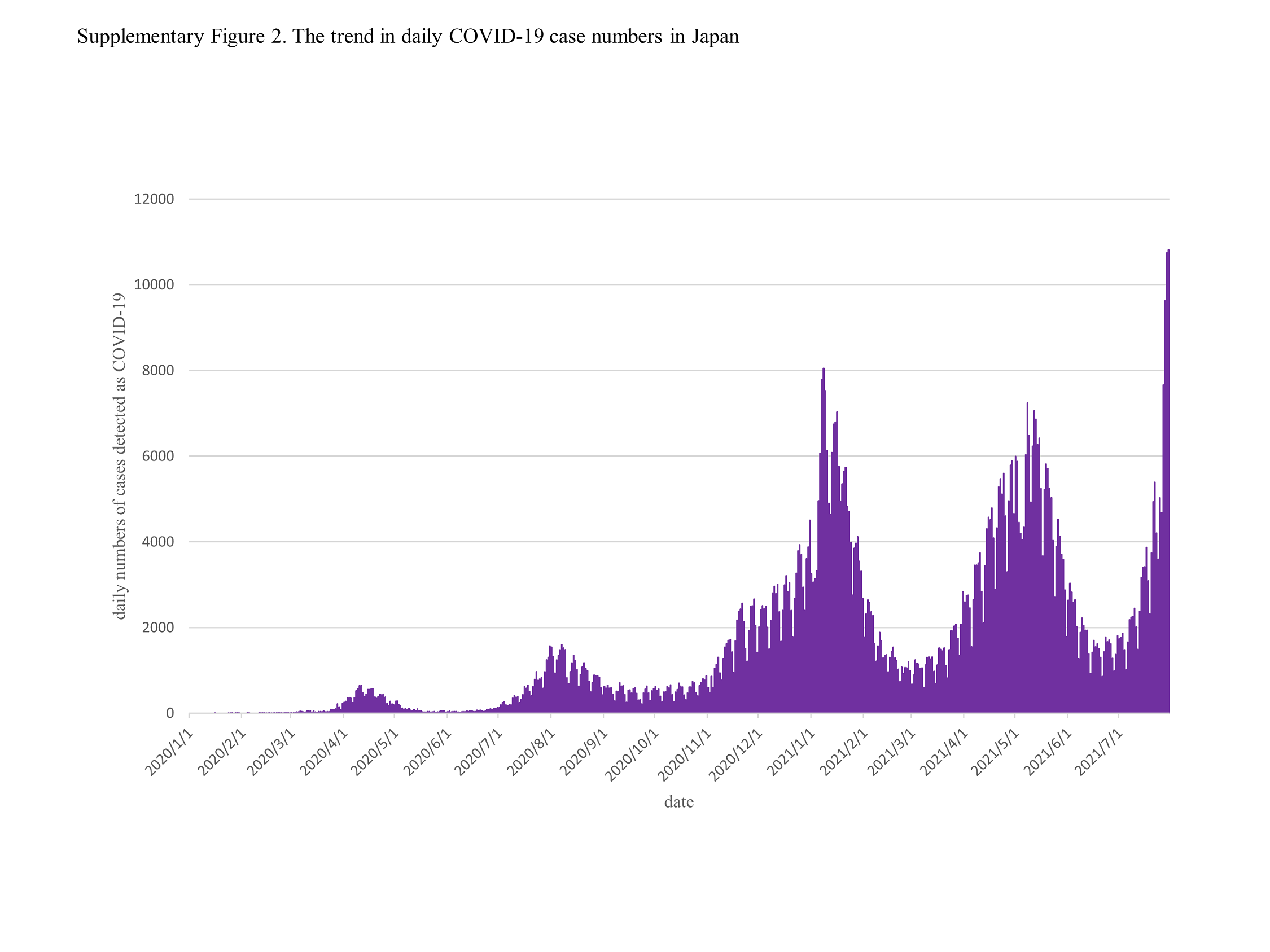

Supplement: S2 Fig — COVID-19, Coronavirus disease 2019. (TIF) [file pone.0273952.s002.TIF]

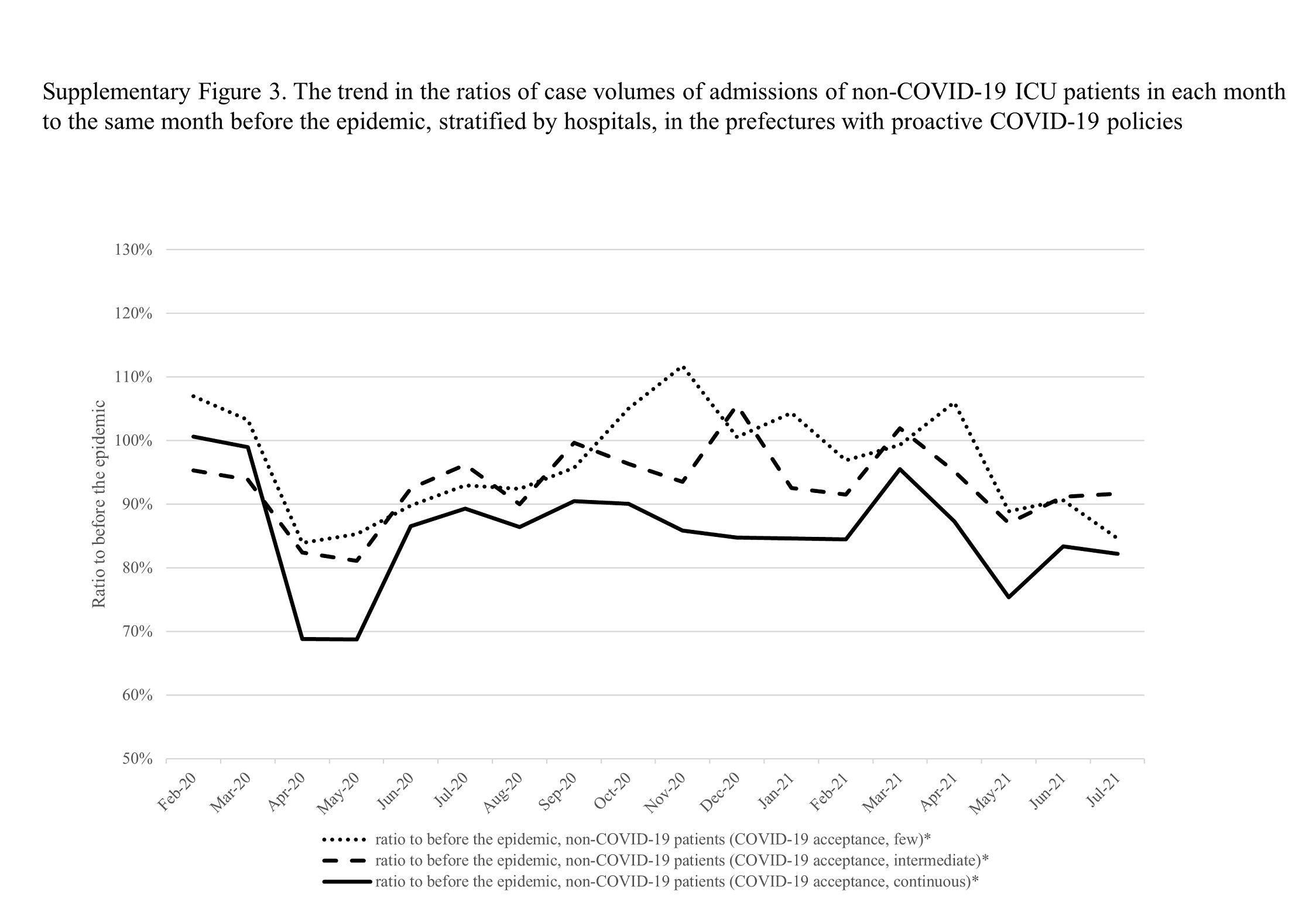

Supplement: S3 Fig — ICU, intensive care unit; COVID-19, Coronavirus disease 2019; * Indicates new admissions to ICU. (TIF) [file pone.0273952.s003.TIF]

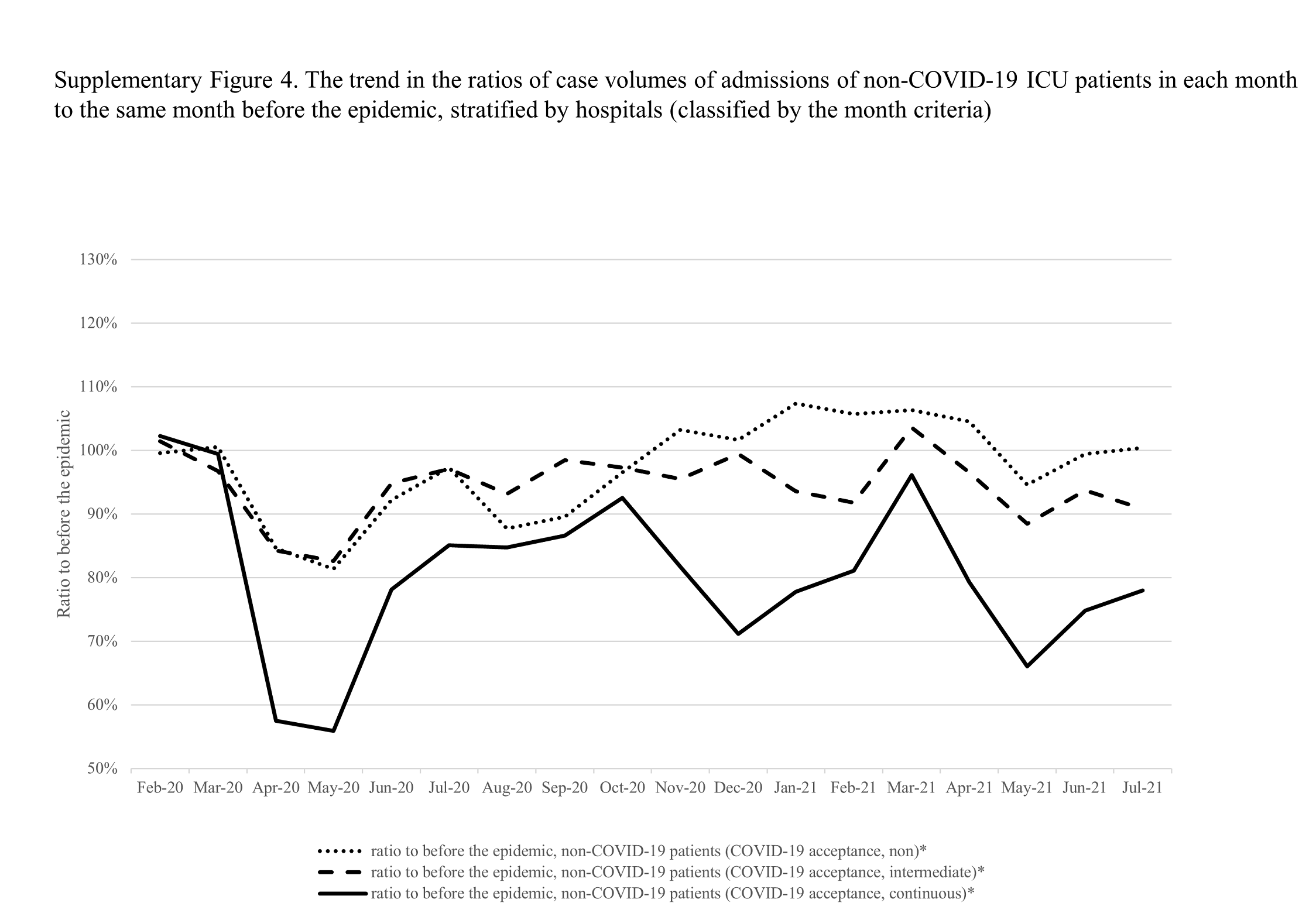

Supplement: S4 Fig — ICU, intensive care unit; COVID-19, Coronavirus disease 2019. * Indicates new admissions to ICU. (TIF) [file pone.0273952.s004.TIF]

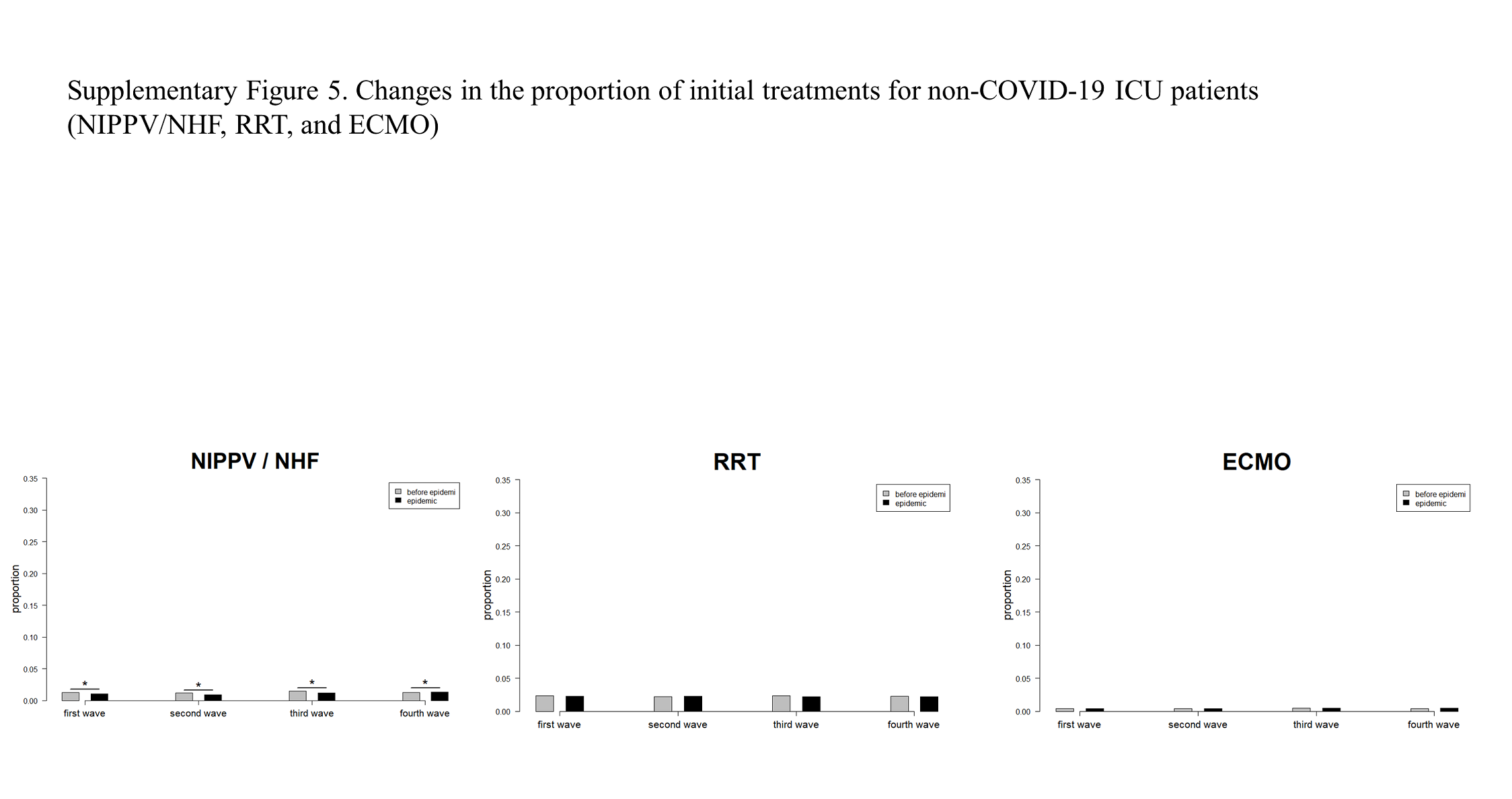

Supplement: S5 Fig — NIPPV, noninvasive positive pressure ventilation; NHF, nasal high flow; RRT, renal replacement therapy; ECMO, extracorporeal membrane oxygenation. * Indicates statistical difference. (TIF) [file pone.0273952.s005.TIF]
