# Supplementary material for: Medium-term impacts of the waves of the COVID-19 epidemic on treatments for non-COVID-19 patients in intensive care units: A retrospective cohort study in Japan
Source: PLoS One. 2022 Sep 26;17(9):e0273952. doi: 10.1371/journal.pone.0273952 (PMC9512181; doi:10.1371/journal.pone.0273952)
Supplement: S1 Table — COVID-19, Coronavirus disease 2019; ICU, intensive care unit; sICU, specialized-care ICU; IMV, invasive mechanical ventilation. * From Feb-20 to Mar-21, ratios of case numbers to those of the same months 1-year before (Feb-19 to Mar-20) are shown and from Apr-21 to Jul-21, ratios of case numbers to those of the same months 2-years before (Apr-19 to Jul-19) are shown. ** Indicates new admissions to ICU. (DOCX) [file pone.0273952.s006.docx]

Supplementary Table 1. Trends in the ratios of case volumes of admissions to ICUs in each month to the corresponding month before the epidemic

|  | Case numbers (ratio to before the epidemic*) | | | | | | |
| --- | --- | --- | --- | --- | --- | --- | --- |
|  | New admitted COVID-19 ICU patients | All patients (patient-days) | All patients** | Non-COVID-19 patients** | Non-COVID-19 patients initially admitted to sICU** | Non-COVID-19 patients initially undergoing IMV** | Non-COVID-19 patients initially administering vasopressor** |
| Feb-20 | 4 | 46273 (102.8%) | 13057 (101.4%) | 13053 (101.4%) | 4452 (101.1%) | 1892 (103.7%) | 3503 (101.4%) |
| Mar-20 | 29 | 45216 (94.8%) | 13232 (97.5%) | 13203 (97.3%) | 4667 (100.2%) | 1806 (101.9%) | 3613 (99.4%) |
| Apr-20 | 315 | 39733 (86.9%) | 11212 (83.1%) | 10897 (80.8%) | 3934 (86.1%) | 1427 (78.0%) | 3140 (90.0%) |
| May-20 | 76 | 38405 (82.3%) | 10572 (79.6%) | 10496 (79.0%) | 3623 (81.1%) | 1280 (73.6%) | 2808 (82.8%) |
| Jun-20 | 17 | 40019 (89.4%) | 12128 (92.4%) | 12111 (92.3%) | 4375 (99.8%) | 1376 (88.7%) | 3263 (97.6%) |
| Jul-20 | 247 | 44401 (97.0%) | 13337 (97.2%) | 13090 (95.4%) | 4599 (96.2%) | 1408 (91.0%) | 3451 (96.8%) |
| Aug-20 | 552 | 46632 (99.6%) | 13078 (95.7%) | 12526 (91.7%) | 4165 (90.9%) | 1400 (85.5%) | 3267 (97.0%) |
| Sep-20 | 217 | 43549 (97.2%) | 12687 (97.9%) | 12470 (96.2%) | 4266 (94.7%) | 1310 (85.1%) | 3223 (103.1%) |
| Oct-20 | 247 | 46554 (98.2%) | 13778 (98.4%) | 13531 (96.6%) | 4561 (94.9%) | 1506 (89.4%) | 3511 (98.9%) |
| Nov-20 | 674 | 48895 (103.1%) | 13603 (99.1%) | 12929 (94.2%) | 4325 (93.7%) | 1635 (89.4%) | 3469 (97.6%) |
| Dec-20 | 900 | 53144 (107.3%) | 14721 (102.1%) | 13821 (95.8%) | 4481 (92.2%) | 1812 (87.9%) | 3758 (94.6%) |
| Jan-21 | 1190 | 57811 (112.3%) | 14640 (100.7%) | 13450 (92.5%) | 4335 (89.6%) | 1880 (83.8%) | 3674 (95.2%) |
| Feb-21 | 516 | 47593 (102.9%) | 12449 (95.3%) | 11933 (91.4%) | 3980 (89.4%) | 1541 (81.4%) | 3360 (95.9%) |
| Mar-21 | 508 | 49696 (109.9%) | 14081 (106.4%) | 13573 (102.8%) | 4580 (98.1%) | 1680 (93.0%) | 3702 (102.5%) |
| Apr-21 | 1252 | 52616 (115.1%) | 14049 (104.2%) | 12797 (94.9%) | 4273 (93.5%) | 1562 (85.4%) | 3535 (101.3%) |
| May-21 | 1712 | 56331 (120.8%) | 13133 (98.8%) | 11421 (86.0%) | 3880 (86.8%) | 1408 (81.0%) | 3072 (90.5%) |
| Jun-21 | 932 | 48110 (107.5%) | 12943 (98.6%) | 12011 (91.5%) | 4189 (95.6%) | 1373 (88.5%) | 3383 (101.2%) |
| Jul-21 | 969 | 46418 (101.4%) | 13279 (96.8%) | 12310 (89.7%) | 4146 (86.7%) | 1366 (88.2%) | 3307 (92.8%) |
| COVID-19, Coronavirus disease 2019; ICU, intensive care unit; sICU, specialized-care ICU; IMV, invasive mechanical ventilation * From Feb-20 to Mar-21, ratios of case numbers to those of the same months 1-year before (Feb-19 to Mar-20) are shown and from Apr-21 to Jul-21, ratios of case numbers to those of the same months 2-years before (Apr-19 to Jul-19) are shown. ** Indicates new admissions to ICU | | | | | | | |
