# Supplementary material for: Medium-term impacts of the waves of the COVID-19 epidemic on treatments for non-COVID-19 patients in intensive care units: A retrospective cohort study in Japan
Source: PLoS One. 2022 Sep 26;17(9):e0273952. doi: 10.1371/journal.pone.0273952 (PMC9512181; doi:10.1371/journal.pone.0273952)
Supplement: S2 Table — COVID-19, Coronavirus disease 2019; ICU, intensive care unit. * From Feb-20 to Mar-21, ratios of case numbers to those of the same months 1-year before (Feb-19 to Mar-20) are shown and from Apr-21 to Jul-21, ratios of case numbers to those of the same months 2-years before (Apr-19 to Jul-19) are shown. ** Indicates new admissions to ICU. (DOCX) [file pone.0273952.s007.docx]

Supplementary Table 2. Trends in the ratios of case volumes of non-COVID-19 patient admissions to ICUs in each month to the same month in the previous year, stratified by hospitals

|  | Case numbers (ratio to before the epidemic*) | | |
| --- | --- | --- | --- |
|  | Non-COVID-19 patients (COVID-19 acceptance, few)** | Non-COVID-19 patients (COVID-19 acceptance, intermediate)** | Non-COVID-19 patients (COVID-19 acceptance, continuous)** |
| Feb-20 | 1816 (104.5%) | 5494 (100.8%) | 5743 (101.0%) |
| Mar-20 | 1773 (94.0%) | 5642 (98.7%) | 5788 (97.1%) |
| Apr-20 | 1700 (87.9%) | 4998 (89.3%) | 4199 (70.5%) |
| May-20 | 1601 (85.6%) | 4772 (86.0%) | 4123 (70.3%) |
| Jun-20 | 1798 (96.5%) | 5280 (94.6%) | 5033 (88.7%) |
| Jul-20 | 1905 (100.2%) | 5623 (98.0%) | 5562 (91.5%) |
| Aug-20 | 1773 (94.8%) | 5439 (93.6%) | 5314 (88.8%) |
| Sep-20 | 1794 (99.1%) | 5441 (100.1%) | 5235 (91.7%) |
| Oct-20 | 2023 (105.4%) | 5794 (97.4%) | 5714 (93.1%) |
| Nov-20 | 1948 (106.0%) | 5580 (95.7%) | 5401 (89.2%) |
| Dec-20 | 2108 (105.7%) | 6116 (101.0%) | 5597 (87.8%) |
| Jan-21 | 2158 (106.0%) | 5749 (93.8%) | 5543 (87.0%) |
| Feb-21 | 1903 (104.8%) | 5092 (92.7%) | 4938 (86.0%) |
| Mar-21 | 2061 (116.2%) | 5799 (102.8%) | 5713 (98.7%) |
| Apr-21 | 2081 (107.6%) | 5470 (97.7%) | 5246 (88.1%) |
| May-21 | 1892 (101.2%) | 4996 (90.0%) | 4533 (77.3%) |
| Jun-21 | 1873 (100.5%) | 5234 (93.7%) | 4904 (86.4%) |
| Jul-21 | 1865 (98.1%) | 5376 (93.7%) | 5069 (83.4%) |
| COVID-19, Coronavirus disease 2019; ICU, intensive care unit * From Feb-20 to Mar-21, ratios of case numbers to those of the same months 1-year before (Feb-19 to Mar-20) are shown and from Apr-21 to Jul-21, ratios of case numbers to those of the same months 2-years before (Apr-19 to Jul-19) are shown. ** Indicates new admissions to ICU | | | |
