# Supplementary material for: Medium-term impacts of the waves of the COVID-19 epidemic on treatments for non-COVID-19 patients in intensive care units: A retrospective cohort study in Japan
Source: PLoS One. 2022 Sep 26;17(9):e0273952. doi: 10.1371/journal.pone.0273952 (PMC9512181; doi:10.1371/journal.pone.0273952)
Supplement: S3 Table — COVID-19, Coronavirus disease 2019; ICU, intensive care unit. * From Feb-20 to Mar-21, ratios of case numbers to those of the same months 1-year before (Feb-19 to Mar-20) are shown and from Apr-21 to Jul-21, ratios of case numbers to those of the same months 2-years before (Apr-19 to Jul-19) are shown. ** Indicates new admissions to ICU. (DOCX) [file pone.0273952.s008.docx]

Supplementary Table 3. Trends in the ratios of case volumes of non-COVID-19 patient admissions to ICUs in each month to the same month in the previous year, stratified by hospitals, in the prefectures with proactive COVID-19 policies

|  | Case numbers (ratio to before the epidemic*) | | |
| --- | --- | --- | --- |
|  | Non-COVID-19 patients (COVID-19 acceptance, few)** | Non-COVID-19 patients (COVID-19 acceptance, intermediate)** | Non-COVID-19 patients (COVID-19 acceptance, continuous)** |
| Feb-20 | 708 (106.9%) | 3025 (95.3%) | 4594 (100.6%) |
| Mar-20 | 723 (103.3%) | 3109 (93.9%) | 4706 (99.0%) |
| Apr-20 | 601 (83.9%) | 2627 (82.4%) | 3299 (68.8%) |
| May-20 | 621 (85.3%) | 2566 (81.1%) | 3252 (68.7%) |
| Jun-20 | 653 (89.8%) | 2936 (92.5%) | 4001 (86.6%) |
| Jul-20 | 691 (93.0%) | 3196 (96.2%) | 4412 (89.3%) |
| Aug-20 | 704 (92.4%) | 3071 (90.0%) | 4185 (86.4%) |
| Sep-20 | 675 (95.7%) | 3103 (99.6%) | 4151 (90.5%) |
| Oct-20 | 768 (105.1%) | 3203 (96.4%) | 4480 (90.1%) |
| Nov-20 | 761 (111.7%) | 3031 (93.5%) | 4210 (85.9%) |
| Dec-20 | 756 (100.5%) | 3486 (105.6%) | 4359 (84.8%) |
| Jan-21 | 817 (104.3%) | 3150 (92.6%) | 4336 (84.6%) |
| Feb-21 | 686 (96.9%) | 2768 (91.5%) | 3880 (84.5%) |
| Mar-21 | 718 (99.3%) | 3170 (102.0%) | 4494 (95.5%) |
| Apr-21 | 759 (106.0%) | 3035 (95.2%) | 4186 (87.3%) |
| May-21 | 647 (88.9%) | 2754 (87.0%) | 3565 (75.4%) |
| Jun-21 | 659 (90.6%) | 2895 (91.2%) | 3855 (83.4%) |
| Jul-21 | 629 (84.7%) | 3044 (91.6%) | 4061 (82.2%) |
| COVID-19, Coronavirus disease 2019; ICU, intensive care unit * From Feb-20 to Mar-21, ratios of case numbers to those of the same months 1-year before (Feb-19 to Mar-20) are shown and from Apr-21 to Jul-21, ratios of case numbers to those of the same months 2-years before (Apr-19 to Jul-19) are shown. ** Indicates new admissions to ICU | | | |
