# Supplementary material for: Medium-term impacts of the waves of the COVID-19 epidemic on treatments for non-COVID-19 patients in intensive care units: A retrospective cohort study in Japan
Source: PLoS One. 2022 Sep 26;17(9):e0273952. doi: 10.1371/journal.pone.0273952 (PMC9512181; doi:10.1371/journal.pone.0273952)
Supplement: S4 Table — COVID-19, Coronavirus disease 2019; ICU, intensive care unit. * From Feb-20 to Mar-21, ratios of case numbers to those of the same months 1-year before (Feb-19 to Mar-20) are shown and from Apr-21 to Jul-21, ratios of case numbers to those of the same months 2-years before (Apr-19 to Jul-19) are shown. ** Indicates new admissions to ICU. (DOCX) [file pone.0273952.s009.docx]

Supplementary Table 4. Trends in the ratios of case volumes of non-COVID-19 patient admissions to ICUs in each month to the same month in the previous year, stratified by hospitals (classified by the month criteria)

|  | Case numbers (ratio to before the epidemic*) | | |
| --- | --- | --- | --- |
|  | Non-COVID-19 patients (COVID-19 acceptance, non)** | Non-COVID-19 patients (COVID-19 acceptance, intermediate)** | Non-COVID-19 patients (COVID-19 acceptance, continuous)** |
| Feb-20 | 968 (99.6%) | 10366 (101.4%) | 1719 (102.3%) |
| Mar-20 | 1000 (100.5%) | 10500 (96.7%) | 1703 (99.4%) |
| Apr-20 | 874 (84.6%) | 9010 (84.3%) | 1013 (57.5%) |
| May-20 | 823 (81.3%) | 8698 (82.6%) | 975 (55.9%) |
| Jun-20 | 930 (92.2%) | 9773 (94.8%) | 1408 (78.1%) |
| Jul-20 | 980 (97.3%) | 10484 (97.1%) | 1626 (85.1%) |
| Aug-20 | 883 (87.7%) | 10151 (93.1%) | 1492 (84.7%) |
| Sep-20 | 858 (89.6%) | 10150 (98.4%) | 1462 (86.6%) |
| Oct-20 | 992 (96.5%) | 10860 (97.3%) | 1679 (92.5%) |
| Nov-20 | 1001 (103.2%) | 10450 (95.5%) | 1478 (81.7%) |
| Dec-20 | 1072 (101.6%) | 11385 (99.4%) | 1364 (71.2%) |
| Jan-21 | 1123 (107.4%) | 10858 (93.6%) | 1469 (77.8%) |
| Feb-21 | 1023 (105.7%) | 9516 (91.8%) | 1394 (81.1%) |
| Mar-21 | 1063 (106.3%) | 10873 (103.6%) | 1637 (96.1%) |
| Apr-21 | 1080 (104.5%) | 10321 (96.5%) | 1396 (79.3%) |
| May-21 | 957 (94.6%) | 9311 (88.4%) | 1153 (66.1%) |
| Jun-21 | 1003 (99.4%) | 9659 (93.7%) | 1349 (74.8%) |
| Jul-21 | 1011 (100.4%) | 9809 (90.8%) | 1490 (78.0%) |
| COVID-19, Coronavirus disease 2019; ICU, intensive care unit * From Feb-20 to Mar-21, ratios of case numbers to those of the same months 1-year before (Feb-19 to Mar-20) are shown and from Apr-21 to Jul-21, ratios of case numbers to those of the same months 2-years before (Apr-19 to Jul-19) are shown. ** Indicates new admissions to ICU | | | |
