# Supplementary material for: Medium-term impacts of the waves of the COVID-19 epidemic on treatments for non-COVID-19 patients in intensive care units: A retrospective cohort study in Japan
Source: PLoS One. 2022 Sep 26;17(9):e0273952. doi: 10.1371/journal.pone.0273952 (PMC9512181; doi:10.1371/journal.pone.0273952)
Supplement: S5 Table — COVID-19, Coronavirus disease 2019; ICU, intensive care unit. P-values show the results of chi-square tests to compare the ratios. (DOCX) [file pone.0273952.s010.docx]

Supplementary Table 5. Statistical analysis to compare hospital categories with the year-over-year changes in non-COVID-19 ICU patients.

| Hospital category | Non-COVID-19 ICU patients in the epidemic (1) | Non-COVID-19 ICU patients in the corresponding months before the epidemic (2) | Ratio ( (1) / (2) ) | P-value |
| --- | --- | --- | --- | --- |
| Classified by the main criteria, in all the prefectures |  |  |  |  |
| COVID-19 acceptance, few | 30,483 | 30,197 | 1.009 | Reference |
| COVID-19 acceptance, intermediate | 86,759 | 91,289 | 0.950 | <.0001 |
| COVID-19 acceptance, continuous | 82,124 | 95,315 | 0.862 | <.0001 |
|  |  |  |  |  |
| Classified by the main criteria, in the prefectures with proactive COVID-19 policies |  |  |  |  |
| COVID-19 acceptance, few | 11,145 | 11,673 | 0.955 | Reference |
| COVID-19 acceptance, intermediate | 48,035 | 51,628 | 0.930 | 0.0783 |
| COVID-19 acceptance, continuous | 64,726 | 77,039 | 0.840 | <.0001 |
|  |  |  |  |  |
| Classified by the month criteria, in all the prefectures |  |  |  |  |
| COVID-19 acceptance, few | 15,673 | 16,154 | 0.970 | Reference |
| COVID-19 acceptance, intermediate | 161,308 | 171,906 | 0.938 | 0.0044 |
| COVID-19 acceptance, continuous | 22,385 | 28,741 | 0.779 | <.0001 |
| COVID-19, Coronavirus disease 2019; ICU, intensive care unit P-values show the results of chi-square tests to compare the ratios. | | | | |
