# Supplementary material for: Medium-term impacts of the waves of the COVID-19 epidemic on treatments for non-COVID-19 patients in intensive care units: A retrospective cohort study in Japan
Source: PLoS One. 2022 Sep 26;17(9):e0273952. doi: 10.1371/journal.pone.0273952 (PMC9512181; doi:10.1371/journal.pone.0273952)
Supplement: S6 Table — OR, odds ratio; CI, confidence interval; ICU, intensive care unit; sICU, specialized-care ICU; eICU, emergency-care ICU; HCU, high care unit; IMV, Invasive Mechanical Ventilation; NIPPV, Noninvasive positive pressure ventilation; NHF, Nasal high flow; ECMO, Extracorporeal membrane oxygenation; RRT, Renal replacement therapy. The ORs were adjusted for the predictors listed in the table. (DOCX) [file pone.0273952.s011.docx]

Supplementary Table 6. Odds ratios of predictors in the prediction model

|  | Adjusted OR (95% CI) | p-value |
| --- | --- | --- |
| sex (male) | 1.317 (1.278-1.358) | <.0001 |
| age |  |  |
| under 45 (reference) | - | - |
| 45-55 | 1.596 (1.438-1.772) | <.0001 |
| 55-65 | 1.980 (1.800-2.179) | <.0001 |
| 65-75 | 2.511 (2.298-2.743) | <.0001 |
| 75-85 | 3.502 (3.211-3.819) | <.0001 |
| 85+ | 5.010 (4.590-5.469) | <.0001 |
| Body mass index |  |  |
| 18.5-25 (reference) | - | - |
| under 18.5 | 1.593 (1.537-1.652) | <.0001 |
| 25-30 | 0.825 (0.790-0.862) | <.0001 |
| 30+ | 0.954 (0.882-1.033) | 0.2496 |
| unmeasured | 2.586 (2.478-2.700) | <.0001 |
| smoking history | 0.853 (0.825-0.882) | <.0001 |
| ICU categories of initial admission |  |  |
| sICU (reference) | - | - |
| eICU | 0.960 (0.915-1.008) | 0.0998 |
| HCU | 0.861 (0.819-0.906) | <.0001 |
| Initial treatment in ICU |  |  |
| IMV | 2.946 (2.845-3.051) | <.0001 |
| NIPPV/NHF | 2.104 (1.933-2.291) | <.0001 |
| RRT | 1.748 (1.634-1.870) | <.0001 |
| ECMO | 14.144 (12.333-16.223) | <.0001 |
| vasopressor | 2.705 (2.616-2.797) | <.0001 |
| Admission process |  |  |
| post emergency operations (reference) | - | - |
| post elective operations | 0.303 (0.285-0.321) | <.0001 |
| medical indication | 2.194 (2.105-2.288) | <.0001 |
| Major Diagnosis Category |  |  |
| Nervous system (reference) | - | - |
| Eye | 0.027 (0.000-36.115) | 0.3252 |
| Ear, nose, mouth and throat | 0.480 (0.353-0.653) | <.0001 |
| Respiratory system | 1.269 (1.209-1.332) | <.0001 |
| Circulatory system | 0.630 (0.602-0.660) | <.0001 |
| Digestive system | 0.878 (0.831-0.928) | <.0001 |
| Musculoskeletal system | 1.059 (0.937-1.198) | 0.3576 |
| Skin and subcutaneous tissue | 0.401 (0.289-0.554) | <.0001 |
| Breast | 1.518 (1.004-2.296) | 0.0479 |
| Endocrine and metabolic system | 0.372 (0.323-0.429) | <.0001 |
| Kidney and urinary system | 0.723 (0.667-0.784) | <.0001 |
| Female reproductive system | 1.242 (0.975-1.581) | 0.0788 |
| Blood and immunological disorders | 2.911 (2.674-3.168) | <.0001 |
| Congenital disease | 0.406 (0.156-1.055) | 0.0643 |
| Pediatric disease | 0.023 (0.000-5777606.185) | 0.7025 |
| Injuries, burns and poisoning | 0.462 (0.431-0.496) | <.0001 |
| Psychiatry | 0.101 (0.046-0.224) | <.0001 |
| Others | 1.639 (1.536-1.749) | <.0001 |
| Unmeasured | 28.858 (26.967-30.883) | <.0001 |
| Month of admission |  |  |
| January | 1.058 (0.995-1.126) | 0.0701 |
| February | 1.041 (0.976-1.110) | 0.226 |
| March | 1.011 (0.948-1.079) | 0.7366 |
| April | 0.942 (0.882-1.006) | 0.0769 |
| May | 0.990 (0.927-1.057) | 0.759 |
| June | 0.930 (0.869-0.995) | 0.0342 |
| July | 0.944 (0.883-1.008) | 0.086 |
| August | 0.959 (0.898-1.024) | 0.2117 |
| September | 0.978 (0.915-1.045) | 0.5059 |
| October | 1.000 (0.937-1.067) | 0.9953 |
| November | 1.048 (0.983-1.117) | 0.152 |
| December (reference) | - | - |
| OR, odds ratio; CI, confidence interval; ICU, intensive care unit; sICU, specialized-care ICU; eICU, emergency-care ICU; HCU, high care unit; IMV, Invasive Mechanical Ventilation; NIPPV, Noninvasive positive pressure ventilation; NHF, Nasal high flow; ECMO, Extracorporeal membrane oxygenation; RRT, Renal replacement therapy  The ORs were adjusted for the predictors listed in the table. | | |
