# Supplementary material for: Medium-term impacts of the waves of the COVID-19 epidemic on treatments for non-COVID-19 patients in intensive care units: A retrospective cohort study in Japan
Source: PLoS One. 2022 Sep 26;17(9):e0273952. doi: 10.1371/journal.pone.0273952 (PMC9512181; doi:10.1371/journal.pone.0273952)
Supplement: S7 Table — SMR, standardized mortality ratio; COVID-19, Coronavirus disease 2019; ICU, intensive care unit; UI, uncertainty interval. * indicates statistically significantly different from 1. (DOCX) [file pone.0273952.s012.docx]

Supplementary Table 7. Standardized mortality in each wave of the epidemic, stratified by hospital categories

| Time period | Hospital category of acceptance of COVID-19 ICU patients | SMR (95% UI) |
| --- | --- | --- |
| First wave (Apr - Jun 2020) | All hospital | 0.990(0.962-1.019) |
|  | Hospitals, continuously accepting COVID-19 ICU patients | 0.961(0.924-0.999)* |
|  | Hospitals, intermediately accepting COVID-19 ICU patients | 1.018(0.981-1.061) |
|  | Hospitals, accepting few COVID-19 ICU patients | 0.987(0.916-1.059) |
| Second wave (Jul - Sep 2020) | All hospital | 0.979(0.953-1.006) |
|  | Hospitals, continuously accepting COVID-19 ICU patients | 0.975(0.942-1.013) |
|  | Hospitals, intermediately accepting COVID-19 ICU patients | 0.956(0.919-0.996)* |
|  | Hospitals, accepting few COVID-19 ICU patients | 1.057(0.992-1.129) |
| Third wave (Oct 2020 - Mar 2021) | All hospital | 0.996(0.980-1.013) |
|  | Hospitals, continuously accepting COVID-19 ICU patients | 1.001(0.977-1.023) |
|  | Hospitals, intermediately accepting COVID-19 ICU patients | 0.984(0.958-1.009) |
|  | Hospitals, accepting few COVID-19 ICU patients | 1.017(0.977-1.061) |
| fourth wave (Apr 2021 - Jul 2021) | All hospital | 0.989(0.964-1.014) |
|  | Hospitals, continuously accepting COVID-19 ICU patients | 0.963(0.933-0.996)* |
|  | Hospitals, intermediately accepting COVID-19 ICU patients | 1.007(0.972-1.044) |
|  | Hospitals, accepting few COVID-19 ICU patients | 1.011(0.948-1.071) |
| SMR, standardized mortality ratio; COVID-19, Coronavirus disease 2019; ICU, intensive care unit; UI, uncertainty inerval * indicates statistically significantly different from 1. | | |
